# Feasibility and Preliminary Outcomes of a Brain/Body Intervention for Postural Tachycardia Syndrome (POTS): Pilot Trial of POts Reprocessing Therapy (PORT)

**DOI:** 10.21203/rs.3.rs-10021161/v1

**Published:** 2026-06-22

**Authors:** Taylor B. Crouch, Spencer Owen Chase, Tammy Redman, Mary Wells, Yoni K. Ashar, Howard Schubiner, Madison Maxwell, Elliot Popoff, Gisela Chelimsky, Thomas Chelimsky

**Affiliations:** Virginia Commonwealth University School of Medicine; Virginia Commonwealth University School of Medicine; Virginia Commonwealth University School of Medicine; Virginia Commonwealth University School of Medicine; University of Colorado Anschutz Medical Campus School of Medicine; Ascension Providence Hospital Southfield Campus; Virginia Commonwealth University School of Medicine; Virginia Commonwealth University School of Medicine; Virginia Commonwealth University School of Medicine; Virginia Commonwealth University School of Medicine

**Keywords:** postural tachycardia syndrome, pain reprocessing therapy, neuroplasticity, nonpharmacological interventions

## Abstract

**Purpose:**

Postural tachycardia syndrome (POTS) is a chronic autonomic disorder characterized by orthostatic intolerance and frequently accompanied by a broad constellation of debilitating symptoms. Emerging evidence suggests that dysregulation within central–autonomic regulatory networks may contribute to symptom persistence. We developed POts Reprocessing Therapy (PORT), a neuroplasticity-informed behavioral intervention adapted from Pain Reprocessing Therapy. This pilot study evaluated the feasibility, acceptability, and preliminary clinical outcomes of PORT for individuals with POTS.

**Methods:**

We conducted a non-randomized, two-arm, open-label pilot feasibility trial. Adults with clinically significant POTS symptoms (Malmö POTS Symptom Score ≥ 42) received eight weekly PORT sessions (*n* = 14). A comparison group of adults with POTS receiving community care while awaiting entry to an autonomic clinic served as controls (*n* = 15). Self-report measures were collected at baseline and 8-week follow-up, including POTS symptom severity (Malmö), symptom catastrophizing, anxiety, depression, pain, fatigue, and quality of life. Feasibility and acceptability were assessed via recruitment, retention, and treatment satisfaction.

**Results:**

PORT demonstrated strong feasibility and acceptability, with 83% retention and strong satisfaction ratings among completers. Participants receiving PORT reported greater symptom improvement compared with community care controls. Mixed ANOVA analyses revealed significant group × time interactions for POTS symptoms (ηp^2^ = 0.283), anxiety (ηp^2^ = 0.363), symptom catastrophizing (ηp^2^ = 0.193), and depression (ηp^2^ = 0.186), reflecting moderate-to-large effect sizes.

**Conclusion:**

This pilot study suggests that PORT is a feasible and acceptable intervention and may be associated with improvements in POTS symptom burden and related psychological processes. Findings provide preliminary support for targeting central–autonomic processes through neuroplasticity-based interventions and warrant larger randomized controlled trials to evaluate efficacy and underlying mechanisms.

## Introduction

Postural tachycardia syndrome (POTS) is a common disorder of the autonomic nervous system (ANS) characterized by chronic orthostatic intolerance and an excessive increase in heart rate upon standing [1].

Patients with POTS also often experience a variety of comorbid, non-orthostatic debilitating symptoms, including fatigue, nausea, headaches, and gastrointestinal problems [1, 2]. POTS disproportionately affects young women and frequently co-occurs with other chronic illnesses such as migraines, irritable bowel syndrome, chronic fatigue syndrome, Ehlers-Danlos syndrome, and fibromyalgia [3, 4]. The disorder has significantly increased in prevalence since the COVID-19 pandemic [5]. Unfortunately, the disorder contributes to significant disability and reduced function for many, the pathophysiology remains obscure, and there are no established curative treatments [1, 6].

Most of the existing research on POTS has focused on structural, peripheral contributors to POTS, such as cardiovascular and immunologic pathophysiological mechanisms [7, 8]. There is clear documentation of multiple structural and physiologic abnormalities in patients with POTS (e.g., hypovolemia, small fiber neuropathy, altered norepinephrine levels), but an etiological role of these abnormalities has not been established. Nonetheless, treatment recommendations for POTS align with the aforementioned peripheral lens, focusing on symptom management based on clinical presentation and, sometimes, presumed subtype. Subtypes include neuropathic, hyperadrenergic, and hypovolemic POTS, often treated with vasoconstrictive agents, beta blockers, and volume expansion, respectively [9]. However, no evidence supports an etiological role for these subtype mechanisms and many patients show characteristics of multiple subtypes, with one study finding that over half of patients displayed features of 2 or 3 subtypes [10]. Across subtypes, there is limited established efficacy of pharmacologic treatments [2].

Historically, less attention was given to central nervous system (CNS) mechanisms in POTS, though this has shifted alongside growing recognition of the inseparability of central and autonomic nervous system processes and peripheral physiological function [11–13]. Increasingly, researchers have proposed that POTS may involve dysfunction within central–autonomic regulatory networks rather than arising solely from peripheral abnormalities [8–10]. This view is supported by evidence of reduced autonomic flexibility (e.g., lower heart rate variability [14] and impaired parasympathetic activity [15, 16]), along with alterations in brain regions involved in autonomic control, emotion regulation, and sensorimotor integration [17]. Emerging work further implicates implicit central learning processes, including heightened interoceptive vigilance and threat-based predictive processing. For example, Norcliffe-Kaufmann et al. [18] found that, compared to healthy controls, patients with POTS had higher somatic vigilance and anticipatory tachycardia *prior* to assuming an upright position. Further, patients with POTS showed higher tachycardia and hyperventilation during standing, both of which were predicted by heightened vigilance and epinephrine release.

Crucially, a CNS-based model of POTS does not negate the clear structural, physiological abnormalities observed in POTS, but proposes that these changes are only “part of the story.” The model posits that a “fuller story” also includes dysregulation of brain-body communications, driven by an altered feedback loop between the CNS, the ANS, and the end-organ, associated with impaired parasympathetic (vagal) regulation and persistent sympathetic activation in the wake of a threat. In this view, POTS may be maintained by maladaptive neurophysiological learning, in which threat-related neural circuits become persistently and inappropriately engaged following an initial precipitating event. Consistent with this theory, research has established that POTS typically develops after a “threat to the system” of some type, such as a virus (e.g., COVID-19), infection, surgery, immunization, head trauma, or psychological trauma [19–21]. To date, there is limited evidence that the specific precipitating trigger of POTS (e.g., infection versus head trauma) gives rise to meaningfully distinct underlying disease mechanisms, inviting ongoing exploration of a unifying pathophysiological framework that can account for heterogeneity in trigger while explaining shared clinical features.

Such a model suggests that POTS may be mechanistically aligned with other centrally-driven chronic conditions, such as fibromyalgia, irritable bowel syndrome, and chronic low back pain, which are increasingly understood to be driven by altered, brain-based threat processing rather than ongoing structural pathology [22–24]. These advances in the neuroscience of pain and interoception have led to the development of neuroplasticity-based interventions that target maladaptive predictive processing and aim to “rewire” the neural pathways that lead the brain to generate pain over time [25]. One such approach, Pain Reprocessing Therapy (PRT), aims to reduce pain by teaching patients to reinterpret bodily sensations as non-dangerous – based on the idea that pain is originally protective but can become “overlearned” in the nervous system over time – and interrupt conditioned threat responses through cognitive and experiential practices. Randomized trials of PRT have demonstrated substantial and durable symptom improvement in chronic pain populations [26, 27].

Despite the strong theoretical alignment between these treatments and proposed mechanisms of POTS, brain/body, neuroplasticity-based interventions have received little empirical investigation in this population. In collaboration with multidisciplinary experts and patients with lived experience, we developed an adapted version of PRT for POTS, called POts Reprocessing Therapy (PORT) [28]. In the current study, we aimed to evaluate the feasibility, acceptability, and preliminary efficacy of this intervention in a pilot trial. We hypothesized that participants receiving PORT would demonstrate clinically meaningful improvements in POTS symptoms and related outcomes, providing preliminary support for a novel treatment approach for this debilitating condition.

## Methods

This study was a non-randomized, two-arm, open-label pilot feasibility trial conducted from July 2024 to August 2025. The study was approved by the Institutional Review Board at Virginia Commonwealth University.

### Participants

Eligibility criteria included the following:

- Self-reported diagnosis of POTS
- Score 42 or higher on the Malmo POTS assessment[1] [29]
- **Intervention group only**: demonstrates orthostatic intolerance in clinic, as evidenced by > 30 bpm heart rate increase upon standing,[2] with < 20 systolic and < 10 diastolic blood pressure decrease
- Symptoms present and affecting functioning for > 3 months
- Age 18 years or older
- Willing and able to participate in a weekly, in-person treatment
- Able to read, write, and speak English

Exclusion criteria were as follows:

- Previously received PORT
- Has a cognitive, physical, or mental disability expected to interfere with ability to consent and/or participation in treatment
- Currently pregnant or incarcerated

### Procedures

Participants were recruited from the Comprehensive Autonomic Clinic (CAC) at VCU, a multidisciplinary clinic treating individuals with autonomic disorders, including POTS, syncope, and orthostatic intolerance (OI). The clinic has a waitlist of approximately 2,000 individuals at any given time. Potential candidates for the PORT condition were identified by CAC clinicians from new (i.e., entering the clinic after being on the waitlist) and existing CAC patients. Participants for the community care (waitlist control) condition were recruited from the CAC waitlist, which consists of individuals awaiting entry to the CAC and presumably engaging in community care and/or self-management in the meantime. Participants were recruited for the community care condition if their position on the waitlist indicated their new patient appointment visit to be scheduled more than 6 months in the future. Prospective participants in both the PORT intervention and community care group were contacted by a clinical research coordinator to offer information regarding study participation and evaluate eligibility via a prescreening form. After confirming eligibility and interest, participants underwent an informed consent process then completed a set of baseline questionnaires via REDCap.

Participants in the PORT intervention group were randomly allocated to one of three treating clinicians using a random number generator. In some instances, logistical reasons led to scheduling with a particular therapist; e.g., if a participant could only complete visits on a particular day/time that did not work for all clinicians. PORT participants were contacted by clinical schedulers to set up their weekly PORT visits with their assigned clinician.

### Interventions

#### PORT.

PORT is a brain/body therapeutic treatment that includes: 1) an initial medical evaluation to assess likely centralized versus structural contributors to POTS using an established approach for identifying centralization of chronic symptoms, provide individualized assessment feedback, and offer initial centralized symptom education for the patient [30]. This was completed by an autonomic neurologist well-versed in this treatment model (T.Ch.); 2) An initial intake and eight 50–60 minute PORT treatment sessions. Sessions were delivered by three health psychologists, all of whom had received formal training in Pain Reprocessing Therapy and were involved in development of PORT (T.Cr., M.W., T.R.). Intervention content includes psychoeducation to reframe POTS symptoms as protective but no longer adaptive “false alarm” signals from the nervous system, mindfulness meditation and somatic inquiry, and graded exposure to symptoms (e.g., with imagined or actual standing) paired with cognitive and somatic regulation strategies. There is also a focus on addressing emotional, coping, and interpersonal factors conceptualized to be contributing to or maintaining symptoms. Patients also complete at-home experiential and written exercises. Therapist adherence to the protocol was monitored by having therapists complete a tracking form after each session in which they identified which key interventions were implemented.

#### Waitlist/Community Care.

Waitlist/CC participants were informed that they would not be receiving active treatment through the CAC as part of the study and should continue engaging in their current/usual treatment or management approach. Baseline assessment confirmed that the majority of participants in both groups reported using a variety of medical and self-management strategies for their POTS (see Results, Table 1). Participation in the study did not affect participants’ waitlist position. The community care condition was not intended to compare outcomes with a defined, alternative treatment model, but rather to help account for potential regression to the mean, demand effects, and/or natural recovery over the study time period, particularly because natural recovery has been documented in POTS [31].

### Measures

Primary outcomes in this study included feasibility and acceptability of the PORT intervention. Preliminary treatment effects were measured as a secondary outcome and included change in self-reported POTS symptoms, fatigue, anxiety symptoms, depression symptoms, symptom cognitions, and quality of life.

#### Baseline Measures.

At baseline, several demographic and medical history background variables were collected. Demographics included age, race, ethnicity, and gender. Medical information included general autonomic dysfunction over the prior year and was assessed with the Composite Autonomic Symptom Score-31 (COMPASS-31), a 31-item measure that assesses autonomic symptom severity in the orthostatic, vasomotor, secretomotor, gastrointestinal, bladder, and pupillomotor domains. Higher scores indicated greater autonomic dysfunction [32]. Treatment history was evaluated by asking participants to indicate whether various POTS treatments had been recommended to them previously, whether the participant had utilized it, and whether it helped/did not help with POTS symptom alleviation. These included a variety of pharmacological and non-pharmacological treatments such as bed rest, IV fluids, beta blockers, physical therapy, salt supplements, practitioner-supported/self-directed behavioral health strategies, yoga, and vagal nerve stimulation.

#### Feasibility and Acceptability.

Feasibility was determined through study recruitment rates, PORT session completion rates, and assessment completion rates. Additionally, therapist adherence to the intervention approach was measured via tracking logs completed after each session to indicate which, if any, key treatment components were completed during the session, as well as if any adverse events occurred. The key treatment components were selected a priori by the research/clinical team and included interventions such as somatic inquiry (split by its 3 components), intentional exposure to symptoms, and expressing and processing of difficult emotions.

Acceptability was measured by treatment satisfaction items completed post-treatment. Items asked participants to rate their perceptions of overall changes symptom and functioning as a result of the intervention, enjoyment and satisfaction, whether they would recommend the treatment to a friend/family member with similar issues, whether they would participate in the treatment again, whether the treatment was too short/long/the right length, and ease of entry into the research study. Participants were also provided the opportunity to provide open-ended feedback about aspects of the intervention they found helpful, unhelpful, or confusing. Adverse events were assessed at each visit using a session tracking from that included recording any adverse events (e.g., fainting) as well as any details on the nature and severity of the event.

#### Preliminary Clinical Outcomes.

POTS symptom burden was measured with the Malmö POTS Symptom Score, a 12-item measure designed to evaluate POTS symptom severity that has been validated alongside tilt-table testing [33]. Scores range from 0 to 120, with higher scores are indicative of greater symptom severity. Sum scores of ≥ 42 are considered clinically significant.

Depression symptoms were assessed with the Center for Epidemiologic Studies Depression Scale (CES-D), a 20-item measure designed to measure frequency of depressive symptoms in the past week on a scale of 0–3 (0 = less than one day in the past week, 3 = most days out of the past week) [34]. Scores of ≥ 16 are considered clinically significant. Anxiety symptoms were measured with the Generalized Anxiety Disorder (GAD-7) scale, a 7-item measure assessing anxiety symptom frequency in the past two weeks on a scale of 0–3 (0 = not at all, 3 = nearly every day), and 1 item assessing functional impacts due to symptoms [35]. Sum scores of 0–4 are indicative of no/minimal anxiety, scores of 5–9 represent mild anxiety, scores of 10–14 represent moderate anxiety, and 15–21 represent severe levels of anxiety. General quality of life was measured with the PROMIS Global Health scale, a 10-item measure assessing quality of life in the physical and mental functioning domains [36].

In addition to POTS symptoms, other physical symptom measures were also included. The Pain, Enjoyment of Life and General Activity (PEG-3) scale is a 3-item questionnaire used to assess pain levels and interference with enjoyment of life/activity and asks participants to rate these constructs on a scale of 0–10 [37]. Average scores of ≥ 7 represent severe impairments to functioning due to pain. The Fatigue Severity Scale (FSS), a 9-item measure designed to assess fatigue as a symptom of various chronic conditions, was used to assess levels of fatigue. Higher scores indicate higher levels of fatigue- sum scores of ≥ 36 are considered clinically significant [38].

The Chronic Symptoms Attribution Scale, a 7-item measure adapted from the Chronic Pain Attribution Scale [39] utilized in PRT clinical trials and tailored towards general autonomic symptom attributions, assessed participant perceptions on whether their symptoms were due to structural issues or brain processes. Higher scores on items indicate a greater level of agreement that symptoms are caused by brain processes. The Symptom Catastrophizing Scale was adapted from the Pain Catastrophizing Scale (PCS), a 13-item measure designed to evaluate levels of catastrophizing- specifically rumination, magnification, and helplessness related to pain and tailored towards POTS symptoms [40]. Higher scores indicate higher levels of catastrophizing; sum scores of > 30 are considered clinically significant.

### Statistical Analyses

All statistical analyses were conducted in SPSS Version 31.0. Missing data patterns were assessed via Little’s MCAR test, and estimation maximization was used to impute data missing completely at random (< 5% of all data). This study was primarily focused on feasibility/acceptability and was not designed to test for statistically significant differences between groups. We followed guidelines for determining pilot study sample size a priori as outlined by Cocks and Torgerson [41], which are designed using 1-sided 80% confidence interval (CI) testing to determine whether the effect is of sufficient magnitude to proceed with a full trial of the intervention (aiming for a sample at least 9% of main trial’s sample size). The sample size required to detect a difference of 15% power in the full trial (with 80% power, alpha = 0.05, two-sided), in repeated measures ANOVA analyses, is 236. Therefore, a pilot sample of 22 would produce a one-sided 90% CI that would exclude finding a 15% difference in the full trial. To account for attrition, we increased the intended sample size by 25%, thereby aiming to recruit a total of 28 participants (14 per group). This sample size exceeds published standards for statistical power in pilot studies [42].

Demographic differences between groups were examined with independent samples *t*-tests for continuous variables, χ^2^ tests for nominal variables, and Fisher’s Exact Test for variables with less than five observations per cell. Clinical outcomes were assessed using 2 (Time: Pre/Post) × 2 (Group: PORT vs Control) analyses of variance (ANOVA). Because pilot studies are not typically powered for definitive hypothesis testing, standardized effect sizes are recommended to aid interpretation and inform future trials. Therefore, Cohen’s d effect sizes were calculated for change scores, representing standardized mean difference between groups, in addition to partial η^2^ effect sizes from the ANOVA models [43, 44]. Effect sizes of 0.01, 0.06, and 0.14 for partial ηp^2^ and 0.20, 0.50, and 0.80 for Cohen’s d, were interpreted as small, medium, and large effects, respectively [43, 45].

## Results

**Figure 1:**
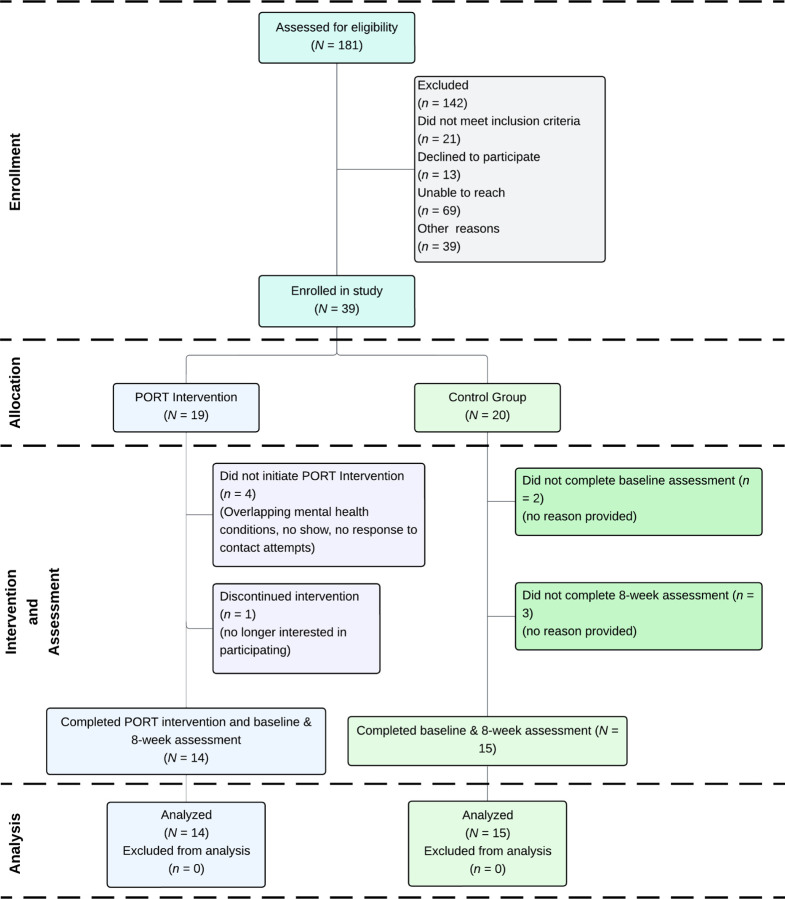
CONSORT flow diagram of the PORT study

A total of 181 prospective participants were screened, 39 completed consent and initial questionnaires (19 PORT participants and 20 community care controls), and 29 completed the study (14 PORT participants and 15 community care controls). See Fig. 1 for CONSORT Flow Diagram. Participant demographics and baseline characteristics are provided in Table 1. The sample was comprised exclusively of women and non-binary individuals. Baseline COMPASS-31 scores indicated substantial autonomic symptom burden in both groups, with values comparable to those reported in other clinical POTS cohorts [32]. Scores on the orthostatic intolerance subscale were similarly elevated, indicating pronounced orthostatic symptoms at study entry. Baseline treatment history indicated that participants were highly treatment-experienced, with most reporting prior use of multiple commonly recommended POTS management strategies, including salt supplementation, exercise, beta-blockers, and physical therapy. This pattern suggests that the sample largely consisted of individuals with persistent symptoms despite engagement in standard clinical POTS management.

### Feasibility and Acceptability Outcomes

Regarding feasibility metrics, of the 19 PORT participants consented, 15 initiated PORT treatment, and 14 completed PORT treatment and all study activities. Four PORT participants did not initiate PORT treatment after the baseline visit due to a self-reported lack of readiness to complete treatment (1), complex and overlapping mental health conditions (1), or they did not show for their first treatment session and did not respond to subsequent contact attempts (2). One participant attended the baseline visit and their first PORT treatment session and chose to end their participation in the study. Of the 20 community care controls consented, 15 completed all study activities. Five participants did not complete study activities and were withdrawn from the study. Two of these participants did not complete their baseline assessment and three participants completed the baseline assessment, but did not complete the 8-week follow up assessment. Therapist tracking logs indicated that the therapists completed an average of 93.51% of the identified key treatment components, indicating strong adherence overall.

Acceptance and satisfaction-related outcomes can be found in Table 2 and indicated overall high levels of treatment satisfaction. Participants were also provided the opportunity to share open-ended feedback, which was primarily positive. For example, one participant shared: “*My symptoms got a lot better during the study, and became a lot easier to pinpoint where my symptoms were coming from before they got too intense*.” Similarly, another participant noted, “*I've become a more comfortable and in tune with my body and can more easily track what’s contributing to or worsening symptoms. My symptoms are less frightening and easier to manage.”* Constructive feedback primarily centered on treatment “dose” and timing; e.g., “*I would definitely recommend to anyone with POTS, but I do wish it was longer/more sessions as I feel we just scratched the surface*.” Another participant shared similar feedback: “*PRT had great techniques to share, but did not feel that there was enough time to develop skills for someone like myself that has deep seated childhood traumas and lifelong POTS. Unlearning how your body has worked your whole life isn’t something that can be done in 8 weeks, and I definitely feel like more support is needed*.”

### Preliminary Clinical Outcomes

**Figure 2:**
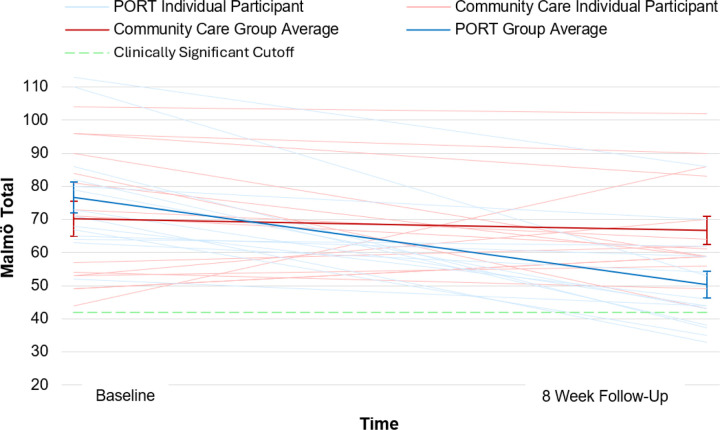
Individual and Group Trajectories in Malmö POTS Symptom Score from baseline to 8-week follow-up

Clinical outcomes assessed at baseline and post-treatment for the two treatment groups are displayed in Table 3. ANOVA time X group outcomes and standardized effect sizes are also displayed to illustrate between-group differences in pre- to post-treatment changes. Figure 2 displays group differences in the primary treatment outcome, POTS symptom severity as measured by the Malmo POTS Symptom Inventory.

## Discussion

In this two-arm pilot trial, we examined the feasibility, acceptability, and preliminary clinical outcomes of PORT, a novel brain-body intervention designed to target maladaptive central-autonomic processes hypothesized to contribute to persistent symptoms in postural tachycardia syndrome (POTS). Overall, the findings support the feasibility and acceptability of this treatment model, with high retention rates and strong patient-reported satisfaction. Preliminary clinical outcomes suggest that PORT may have meaningful potential to reduce POTS symptom burden and several related psychological processes.

Participants who underwent PORT demonstrated substantial reductions in overall POTS symptom severity compared with the community care control group over the same time period, with a large effect size observed for change in Malmö POTS Symptom Score. Improvements were also observed in anxiety symptoms, depressive symptoms, and symptom catastrophizing. Notably, several observed effect sizes were relatively large compared with those commonly reported in psychological intervention trials for chronic medical conditions. While encouraging, these findings should be interpreted cautiously given the pilot nature of the study and the small sample size. Pilot trials are not designed to establish efficacy, and effect sizes observed in early studies are often unstable and may overestimate true treatment effects. Nonetheless, the pattern of change across multiple symptom domains suggests that the intervention warrants further investigation in a larger randomized clinical trial.

The present findings provide preliminary support for the conceptual model underlying PORT, which proposes that persistent symptoms in POTS may reflect, at least in part, maladaptive brain–body signaling within central–autonomic regulatory networks [28]. Within this framework, heightened interoceptive vigilance, conditioned physiological responses, and threat-related predictive processing may contribute to sustained sympathetic activation and symptom persistence. PORT seeks to modify these processes by helping patients reinterpret bodily sensations as non-dangerous, reduce threat-based predictive processing, enhance autonomic self-regulation techniques, and gradually re-engage with previously avoided bodily states and activities. Improvements observed in symptom catastrophizing and anxiety may reflect early changes in these proposed mechanisms. Given that a majority of participants in both groups reported prior use of multiple commonly recommended POTS management strategies, these findings highlight the importance of effective treatment approaches for patients whose symptoms remain burdensome despite conventional management strategies.

Importantly, patient-reported satisfaction was high overall, and the treatment model appeared to resonate with many participants. Patients with POTS frequently report experiences of diagnostic uncertainty, dismissal, or confusion regarding the origin of their symptoms [46]. Providing an explanatory framework that integrates biological and brain-based processes without incorrectly reducing symptoms to psychological causes appeared to be a meaningful component of the intervention. Many participants reported feeling validated within the treatment model and expressed openness to developing skills aimed at regulating brain–body processes contributing to their symptoms. These qualitative impressions are consistent with growing recognition that moving beyond traditional mind–body dualism may be particularly important for conditions involving dysregulation of central, autonomic, and interoceptive systems.

While POTS symptoms themselves improved, several outcomes did not demonstrate meaningful change during the study period. In particular, fatigue severity, pain-related outcomes, and general quality of life remained largely stable across the intervention period. These findings may reflect several possibilities. First, these symptoms may be influenced by additional physiological or behavioral factors not directly targeted by the intervention. Given the focus of this intervention on POTS and early testing phase, the treatment intentionally focused on these symptoms as the primary intervention target, though future research should investigate whether multiple physical symptoms may be target concurrently, e.g., by integrating PRT with PORT. Second, improvements in autonomic symptom perception may precede changes in broader physiological and functional outcomes, which may require a longer period of behavioral adaptation and recovery. Third, the intervention dose may have been insufficient to meaningfully affect these domains in this small pilot sample. Future studies will be needed to clarify whether these symptoms respond to PORT with longer follow-up periods, additional treatment components, or in combination with other rehabilitation approaches.

### Limitations and Future Directions

Several limitations of this study should be considered when interpreting these findings. First, the study employed a small sample size typical of pilot feasibility trials, which limits statistical power and the stability of effect size estimates. Second, the trial used a non-randomized design and an open-label intervention, introducing potential sources of bias including expectancy effects and differences between groups that were not controlled through randomization. Third, the comparison group represented community care rather than a structured active treatment condition, limiting conclusions about the relative efficacy of PORT compared with other behavioral or medical interventions. Still, participants in both groups reported using a variety of other medical and self-management approaches for managing their POTS, so this group may represent reasonably approximate community care as usual. Participants in both groups also continued using a range of medical and self-management strategies for POTS, which were not controlled within the study. Finally, outcomes were measured over a relatively short time period, and the durability of treatment effects remains unknown.

Despite these limitations, the study provides an important first step in evaluating a novel treatment approach for POTS that targets brain–body processes implicated in symptom persistence. The results support the feasibility of delivering PORT within a clinical setting and provide preliminary evidence suggesting that neuroplasticity-based interventions may represent a promising avenue for addressing symptoms that remain difficult to treat with existing medical approaches. Future research will prioritize randomized controlled trials with larger samples to more rigorously evaluate the efficacy of PORT. Such trials will include longer follow-up periods to assess durability of symptom changes and incorporate mechanistic measures, including physiological and neuroimaging indices, to test hypothesized changes in central–autonomic regulation. Understanding which patients benefit most from this intervention and identifying the mechanisms through which symptom improvement occurs will be essential for refining and optimizing this treatment model.

## Conclusion

Traditional biomedical frameworks have often been interpreted through a dualistic distinction between “organic” and “psychological” or “mental” contributors to illness, a distinction that is increasingly at odds with contemporary neuroscience and psychophysiology. For patients with POTS, this dualism can be particularly harmful: the absence of structural cardiac pathology can contribute to symptoms being dismissed as anxiety-related or “all in one’s head,” which is associated with substantial diagnostic delay and experiences of invalidation [46] — despite clear objective autonomic abnormalities. This dualism is further harmful in that it can limit the uptake of mind–body interventions that may directly target symptom-maintaining mechanisms, as both patients and providers may equate such approaches with symptom dismissal rather than physiologically-informed treatment. Indeed, extensive research has shown that mind/body treatments can have positive impacts on objective markers of both CNS [47] and ANS [48] functioning, and more broadly, on a variety of health and disease outcomes [49, 50].

This pilot trial demonstrates that PORT is a feasible and acceptable intervention for individuals with POTS and provides preliminary evidence suggesting potential benefits across multiple symptom domains. While the findings should be interpreted cautiously, they highlight the potential value of integrative brain–body treatment approaches that explicitly target central–autonomic processes in chronic autonomic disorders. Larger randomized trials are needed to determine the efficacy of this intervention and to further clarify the mechanisms through which symptom improvement may occur.

## Figures and Tables

**Table 1:** Demographic and Baseline Medical Characteristics of Sample

	PORT Intervention		Community Care	
	(*n* = 14)		(*n* = 15)	
*Demographics*	n (%)	*M* (SD)	n (%)	*M* (SD)
Age		*28* (12.6)		*33* (9.8)
Sex Assigned at Birth				
Male	1 (7.1)		0	
Female	13 (92.9)		15 (100.0)	
Self-Identified Gender				
Woman	13 (92.9)		14 (93.3)	
Man	0		0	
Non-binary	1 (7.1)		1 (6.7)	
Ethnicity				
Hispanic, Latino, or Spanish Origin	0		1 (6.7)	
Not Hispanic or Latino	14 (100.0)		14 (93.3)	
Race				
American Indian or Alaska Native	1 (7.1)		0	
Asian	0		1 (6.7)	
Black or African American	2 (14.3)		3 (20.0)	
Native Hawaiian or Other Pacific Islander	0		0	
White	12 (85.7)		12 (80.0)	
Middle Eastern or North African	1 (7.1)		1 (6.7)	
*Medical Characteristics*				
COMPASS-31 Total		*56* (10.6)		*53* (9.7)
Orthostatic Intolerance Subscale		*28.6* (8.7)		*26.4* (6.7)
Other Treatments Tried for POTS				
IV Fluids	6 (42.9)		7 (50.0)	
Physical Therapy	10 (71.4)		7 (50.0)	
Salt Supplementation	14 (100.0)		10 (71.4)	
Vagal Nerve Stimulation	1 (7.1)		2 (14.3)	
Exercise	11 (78.6)		12 (85.7)	
Medications				
Beta-Blocker	11 (78.6)		12 (85.7)	
Ivabradine	3 (21.4)		2 (14.3)	
Midodrine	2 (14.3)		4 (28.6)	
Pyridostigmine	4 (28.6)		3 (21.4)	

**Table 2:** PORT Satisfaction Survey Post Intervention

As a result of the PORT Treatment)	Completely agree	Slightly agree	Neither agree nor disagree	Slightly disagree	Completely Disagree
I am satisfied with the PORT treatment overall	9/14 (64.29%)	4/14 (28.57%)	1/14 (7.14%)	0/14 (0.00%)	0/14 (0.00%)
I would recommend the PORT treatment to a family member or friend who has similar problems	10/14 (71.43%)	2/14 (14.29%)	2/14 (14.29%)	0/14 (0.00%)	0/14 (0.00%)
Knowing what I know now, if I could go back I would still choose to participate in the PORT treatment	12/14 (85.71%)	2/14 (14.29%)	0/14 (0.00%)	0/14 (0.00%)	0/14 (0.00%)

**Table 3:** Outcomes Pre- and Post-Treatment

	PORT Intervention (*n*=14)		Community Care (*n* = 15)					
	Baseline	Post	Baseline	Post	Group X Time			Change
	*M* (SD)	*M* (SD)	*M* (SD)	*M* (SD)	*F*	*p*	*np* ^*2*^	*Cohen'sd* ^1^
Malmo	*77* (17.2)	*50* (14.9)	*70* (20.3)	*67* (16.3)	*10.671*	*0.003*	*0.283*	*−1.217*
GAD-7^2^	*13* (5.8)	*8* (4.0)	*6* (3.7)	*10* (5.2)	*14.642*	*< 0.001*	*0.352*	*−1.424*
FSS^3^	*55* (9.0)	*53* (7.2)	*51* (13.5)	*49* (13.8)	*0.045*	*0.833*	*0.002*	*0.079*
CES-D^4^	*30* (12.0)	*24* (8.6)	*18* (12.4)	*20* (12.1)	*6.168*	*0.020*	*0.186*	*−0.921*
SCS^5^	*30* (14.2)	*18* (11.1)	*23* (15.6)	*19* (14.7)	*6.452*	*0.017*	*0.193*	*−0.938*
PEG-3^6^	*6* (1.9)	*5* (1.8)	*5* (1.8)	*4* (1.9)	*0.520*	*0.477*	*0.019*	*−0.268*
PROMIS^7^ Mental	*11* (2.4)	*11* (2.0)	*12* (1.9)	*11* (1.9)	*5.027*	*0.033*	*0.157*	*0.829*
PROMIS Physical	*14* (1.8)	*13* (2.0)	*14* (2.5)	*13* (2.2)	*0.915*	*0.347*	*0.033*	*0.357*

1 Interpretation of Effect Sizes: Cohen’s d effect sizes of 0.20, 0.50, and 0.80, and partial ηp^2^ effect sizes of 0.01, 0.06, and 0.14, were interpreted as small, medium, and large effects, respectively

2 Generalized Anxiety Disorder-7

3 Fatigue Severity Scale

4 Center for Epidemiologic Studies Depression Scale

5 Symptom Catastrophizing Scale

6 Pain, Enjoyment of Life, and General Activity 3-item Questionnaire

7 Patient-Reported Outcomes Measurement Information System
